# The spectrum of movement disorders in young children with *ARX* ‐related epilepsy‐dyskinesia syndrome

**DOI:** 10.1002/acn3.52055

**Published:** 2024-05-06

**Authors:** Shyam K. Akula, Vicente Quiroz, Alissa M. D'Gama, Michelle Y. Chiu, Hyun Yong Koh, Afshin Saffari, Zainab Zaman, Amy Tam, Rasha Srouji, Rozalia Valentine, Kimberly Wiltrout, Anna Pinto, Chellamani Harini, Phillip L. Pearl, Annapurna Poduri, Darius Ebrahimi‐Fakhari

**Affiliations:** ^1^ Movement Disorders Program, Department of Neurology, Boston Children's Hospital Harvard Medical School Boston Massachusetts USA; ^2^ Division of Genetics and Genomics, Boston Children's Hospital Harvard Medical School Boston Massachusetts 02115 USA; ^3^ Division of Epilepsy and Clinical Neurophysiology, Department of Neurology, Boston Children's Hospital Harvard Medical School Boston Massachusetts USA; ^4^ Division of Child Neurology and Inherited Metabolic Diseases Heidelberg University Hospital Heidelberg Germany

## Abstract

Children with developmental and epileptic encephalopathies often present with co‐occurring dyskinesias. Pathogenic variants in *ARX* cause a pleomorphic syndrome that includes infantile epilepsy with a variety of movement disorders ranging from focal hand dystonia to generalized dystonia with frequent status dystonicus. In this report, we present three patients with severe movement disorders as part of *ARX*‐associated epilepsy‐dyskinesia syndrome, including a patient with a novel pathogenic missense variant (p.R371G). These cases illustrate diagnostic and management challenges of *ARX‐*related disorder and shed light on broader challenges concerning epilepsy‐dyskinesia syndromes.

## Introduction

Genetic developmental and epileptic encephalopathies (DEEs) are characterized by infantile onset refractory epilepsy and global developmental delay. While seizures are classically the central feature of DEEs, careful clinical evaluation has broadened the phenotype of many to include hyperkinetic movement disorders.^1^ The phenotypic and molecular spectrum of epilepsy‐dyskinesia syndromes, however, remains incompletely understood.

A key example of an epilepsy‐dyskinesia syndrome that exemplifies diagnostic and management challenges is *ARX‐*related disorder. *ARX* (Aristaless‐related Homeobox) encodes a transcription factor critical for normal forebrain development and for interneuron migration and maturation, among other roles.^2^, ^3^, ^4^ It consists of a paired‐like homeodomain, a C‐terminal Aristaless domain, an octapeptide domain, three nuclear localization sequences, four polyalanine tracts, and a central acidic domain.^5^ Pathogenic variants in *ARX* have a striking genotype–phenotype correlation, with loss‐of‐function alleles leading to neurodevelopmental phenotypes, for example, X‐linked lissencephaly with abnormal genitalia (XLAG), whereas missense variants outside of the homeodomain and polyalanine tract expansions generally lead to a broad range of DEE/epilepsy‐dyskinesia phenotypes with grossly normal cortical brain structure, such as early‐infantile developmental and epileptic encephalopathy, infantile spasms, or myoclonic epilepsy.^6^ While epilepsy and intellectual disability are invariably severe, the movement disorders seen in *ARX* patients range from focal hand dystonia to non‐epileptic myoclonus, spastic diplegia, or generalized dystonia with episodes of status dystonicus.^7^, ^8^

We discuss three unpublished patients with *ARX‐*related epilepsy‐dyskinesia syndrome, including one with a novel *ARX* missense variant in the homeodomain that breaks the traditional pattern of *ARX* genotype–phenotype correlations. We summarize diagnostic challenges and movement disorder manifestations associated with each case. We then provide recommendations for when to consider *ARX‐*related disorder diagnostically and briefly discuss management of dyskinesia specific to the DEE setting.

## Methods

Patients were evaluated at Boston Children's Hospital. Permission for sharing video recordings was obtained. Sequencing was conducted clinically using a multigene panel for Patient #1. Patients #2 and #3 underwent trio rapid genome sequencing through the Gene‐STEPS study.^9^

## Results

### Patient #1

**Figure 1:**
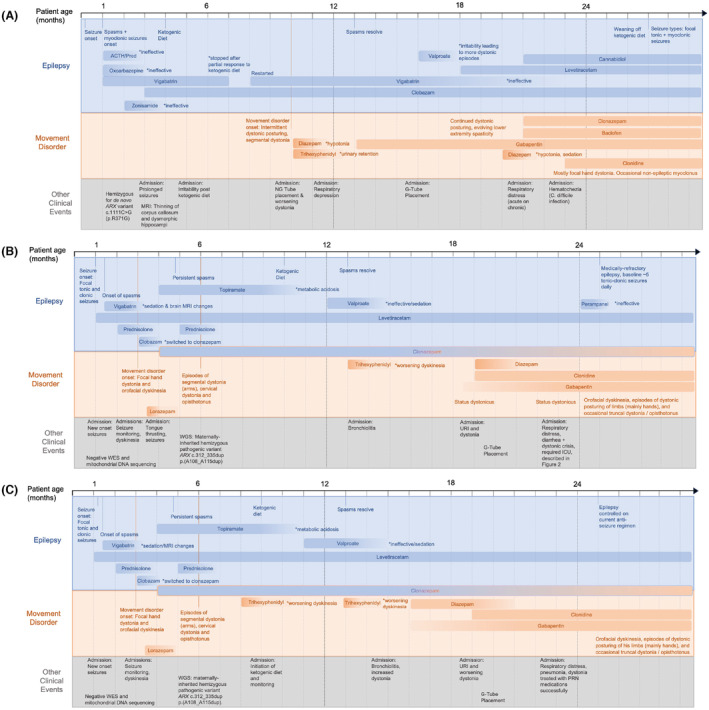
Clinical course of (A) Patient #1, (B) Patient #2, and (C) Patient #3 shows the evolution of a severe developmental and epileptic encephalopathy along with dystonia which includes prominent hand dystonia, in addition to intermittent segmental dystonia of the limbs, and generalized dystonia including status dystonicus with stressors. Orofacial dyskinesia were prominent in Patients #2 and #3. Hypotonia was profound in all three cases. Patient #1 also presented with an evolving spastic paraparesis with pyramidal signs.

Patient #1 is a 2‐year‐old male born at 40 weeks gestation following an uncomplicated pregnancy with a birth weight of 3.03 kg. Seizure onset was at 4 days of age (Fig. 1A). The predominant seizure types included myoclonic seizures (Fig. S1A), which occurred in clusters as well as sporadically, and focal tonic seizures. Seizure types evolved over the first months of life including epileptic spasms and sequential myoclonic‐tonic–clonic seizures. His seizures showed partial response to a combination of cannabidiol, clobazam, levetiracetam, and ketogenic diet (KD). Brain MRI showed marked thinning of the corpus callosum with thickening of the fornices. A multigene epilepsy panel identified a hemizygous *de novo* missense variant in the homeodomain of *ARX* (NM_139058.3: c.1111C >G, p.R371G) which, although not reported previously, results in a likely damaging change in the homeodomain. Unlike other patients with variants in the homeodomain, Patient #1 does not show the severe cortical brain malformations typical for XLAG. From a motor standpoint, the patient presented with profound axial hypotonia and weakness, severe developmental delay, and dysphagia with aspiration. At 10 months of age, he developed intermittent focal and segmental dystonia, involving mostly the distal extremities, including prominent hand dystonia (Fig. 1A). With stressors, such as intercurrent infections or periods of exacerbated seizures, his dystonia worsened (Video S1), requiring hospitalization and treatment with higher doses of clonidine and diazepam. In addition to dystonia, he was found to have generalized non‐epileptic myoclonus (Fig. S1B). His dystonia improved but remained incompletely controlled by a combination of clonidine, baclofen, clonazepam, and gabapentin (Fig. 1A). At the last follow‐up, he continued to have intermittent dystonic posture of the hands, as well as severe hypotonia (Video S2) and global developmental delay.

### Patients #2 and #3

Patients #2 and #3 are twin males born after an uncomplicated monoamniotic‐dichorionic pregnancy at 36 gestational weeks with birth weights of 2.94 and 2.90 kg respectively. The neonatal period was remarkable for significant hypotonia with poor feeding; at 4 weeks, both infants developed seizures, consisting of clusters of tonic seizures followed by epileptic spasms, and focal clonic seizures. EEG at this time showed clusters of generalized epileptic spasms, high voltage background slowing, and abundant multifocal sharp waves (Figs. S2 and S3). Focal seizures were refractory to phenobarbital and levetiracetam and both focal seizures and epileptic spasms showed initial reduction in frequency with vigabatrin. Brain MRI was unremarkable except findings secondary to vigabatrin treatment. Other investigations including metabolic screening (lactic acid, ammonia, amino acids, urine organic acids, carnitine, VLCFA, and CSF profile) were all normal. Rapid exome sequencing and mitochondrial DNA sequencing at 6 weeks of age were nondiagnostic. During admission for seizure management at 12 weeks of age, both twins also developed multiple episodes of abnormal limb and mouth movements (orolingual dystonia and facial dyskinesia) without electrographic correlate (Video S3). Development of these dyskinesias, in addition to prominent hand dystonia and appendicular chorea (Fig. 1B,C, Videos S4 and S5) further suggested a genetic etiology. Whole‐genome testing, performed on a research basis with clinical confirmation, revealed that both brother shared a maternally inherited hemizygous X‐linked pathogenic polyalanine tract expansion in exon 2 of the *ARX* gene (NM_139058.3:c.312‐335dup, p.A108_A115dup).

At 19 months of age, Patient #2 was admitted due to acute hypoxemic respiratory failure attributed to viral pneumonia (Fig. 1B). His parents reported that before admission they noticed worsening of dystonia (Video S6) with episodes that proved unresponsive to repositioning or rescue medications (clonazepam and clonidine). Over the next few days, his dystonic episodes increased in frequency and were accompanied by tachycardia, diaphoresis, and dehydration requiring multiple attempts at rescue medications and increase in dosage of baseline medications (clonidine, clonazepam, diazepam, and gabapentin). Over the next several months, he experienced multiple episodes of generalized dystonia in the setting of infections. At 23 months of age, Patient #2 presented with diarrhea and upper respiratory tract infection with worsening dystonia that showed no response to rescue medications, compatible with an episode of status dystonicus, requiring admission to the intensive care unit. Peak creatine kinase (CK) levels reached 1142 IU/L (reference range 4–175 IU/L). Treatment included clonazepam, diazepam, and a dexmedetomidine infusion. Clinical stability was achieved after 10 days.

At last follow‐up, at age 29 months, both boys showed orofacial dyskinesia (with prominent lingual dystonia), episodes of dystonic posturing of their limbs, in particular the hands, and truncal dystonia, on a background of profound hypotonia. Treatment for dystonia consisted of clonazepam, diazepam, clonidine, and gabapentin. Trihexyphenidyl worsened orofacial dyskinesia. Seizures showed partial response to a combination of KD and levetiracetam, with residual seizures consisting of tonic, clonic, tonic–clonic, myoclonic seizures, and hypomotor seizures. The clinical course was further notable for severe global developmental delay and dysphagia with gastrostomy tube dependence.

## Discussion

### Diagnosis of ARX‐related disorder and the spectrum of dyskinesia associated with ARX variants

Exome/genome sequencing or next‐generation sequencing‐based multigene panels are first‐line genetic tests for patients with suspected neurogenetic conditions because of their relatively high diagnostic yield.^10^ However, in the context of *ARX*‐related disorder, panel and exome sequencing may fail to provide the diagnosis because the most common type of *ARX* disease‐causing variants, polyalanine tract expansions, can be missed. This is due to multiple mechanisms such as suboptimal polymerase processivity and fidelity at repetitive sequences,^11^ as was the case in Patient #2 and #3. WGS and single‐gene testing for *ARX* include a dedicated evaluation for the polyalanine tract expansion and should be considered alongside other genetic investigations for male infants with epilepsy‐dyskinesia syndromes, male infants with epileptic spasms in the setting of agenesis of the corpus callosum with or without ambiguous genitalia and in the association between hand dystonia and epilepsy in infancy or early childhood, which is considered highly suggestive of *ARX*‐related disorder and should prompt specific genetic testing.^12^, ^13^ We here expand this phenotypic spectrum to include the combination of orolingual dystonia, facial dyskinesia, and hand dystonia in the setting of a developmental and epileptic encephalopathy with medically refractory seizures.

### Management of movement disorders in epilepsy‐dyskinesia syndromes

Differentiating between motor seizures and dyskinesia is critical for appropriate management. Distinguishing between these entities is often achieved with video‐EEG and is particularly important in patients with genetic epilepsy‐dyskinesia syndromes who have both seizures and dyskinesia; and at times one symptom may affect quality‐of‐life and require more treatment adjustments than the other.

Treatment should aim at maximizing quality‐of‐life, which may require frequent reassessment and dose adjustments based on clinical status and potential side effects. Patient #2 presented with frequent admissions due to infections with worsening of the dystonic posturing during those episodes. GABA and alpha‐2 adrenergic agonists provided the greatest benefit. Benzodiazepines had to be titrated slowly with close monitoring due to profound concurrent hypotonia resulting in tenuous respiratory status and difficulties managing secretions.

Uncontrolled dystonia may lead to a life‐threatening scenario known as status dystonicus, characterized by frequent and severe episodes of generalized dystonia, often requiring intensive care management, and triggered most frequently by infections.^14^, ^15^ Status dystonicus has been described within the *ARX*‐related disorder,^8^ and in our patients required close monitoring, addressing precipitating factors, initiating supportive measures and sedatives, and several dystonia‐targeted medications. Although currently there are limited data on globus pallidus deep brain stimulation in epilepsy‐dyskinesia syndromes, this intervention should be considered. A multidisciplinary approach and individualized management plans for crises (e.g., a dystonia action plan) can improve outcomes.

## Author Contributions

Designing the research study: A.P. and D.E.F.; acquiring data: all authors; analyzing data: S.K.A, V.Q., A.M.D.G, H.Y.K., A.P., and D.E.F.; writing the manuscript: S.K.A., V.Q., and D.E.F.; revising the manuscript: all authors.

## Conflict of Interest

Research in the Ebrahimi‐Fakhari Laboratory is supported by the Spastic Paraplegia Foundation, the Boston Children's Hospital Translational Research Program, and the National Institutes of Health/National Institute of Neurological Disorders and Stroke (K08NS123552‐01). Vicente Quiroz is supported by a fellowship from the International Parkinson and Movement Disorder Society. Afshin Saffari was funded by the Deutsche Forschungsgemeinschaft (DFG, German Research Foundation) – 448402208. Annapurna Poduri received support from the One8 Foundation. The authors have no conflicts of interest to report.

## Supporting information


Figure S1.

Figure S2.

Figure S3.

Video S1.

Video S2.

Video S3.

Video S4.

Video S5.

Video S6.
