# Supplementary material for: The spectrum of movement disorders in young children with ARX ‐related epilepsy‐dyskinesia syndrome
Source: Ann Clin Transl Neurol. 2024 May 6;11(6):1643–7. doi: 10.1002/acn3.52055 (PMC11187834; doi:10.1002/acn3.52055)
Supplement: Supplementary file 1 — Figure S1. [file ACN3-11-1643-s002.pdf]

Supplementary Figure 1 – Patient 1 Representative EEG Panels

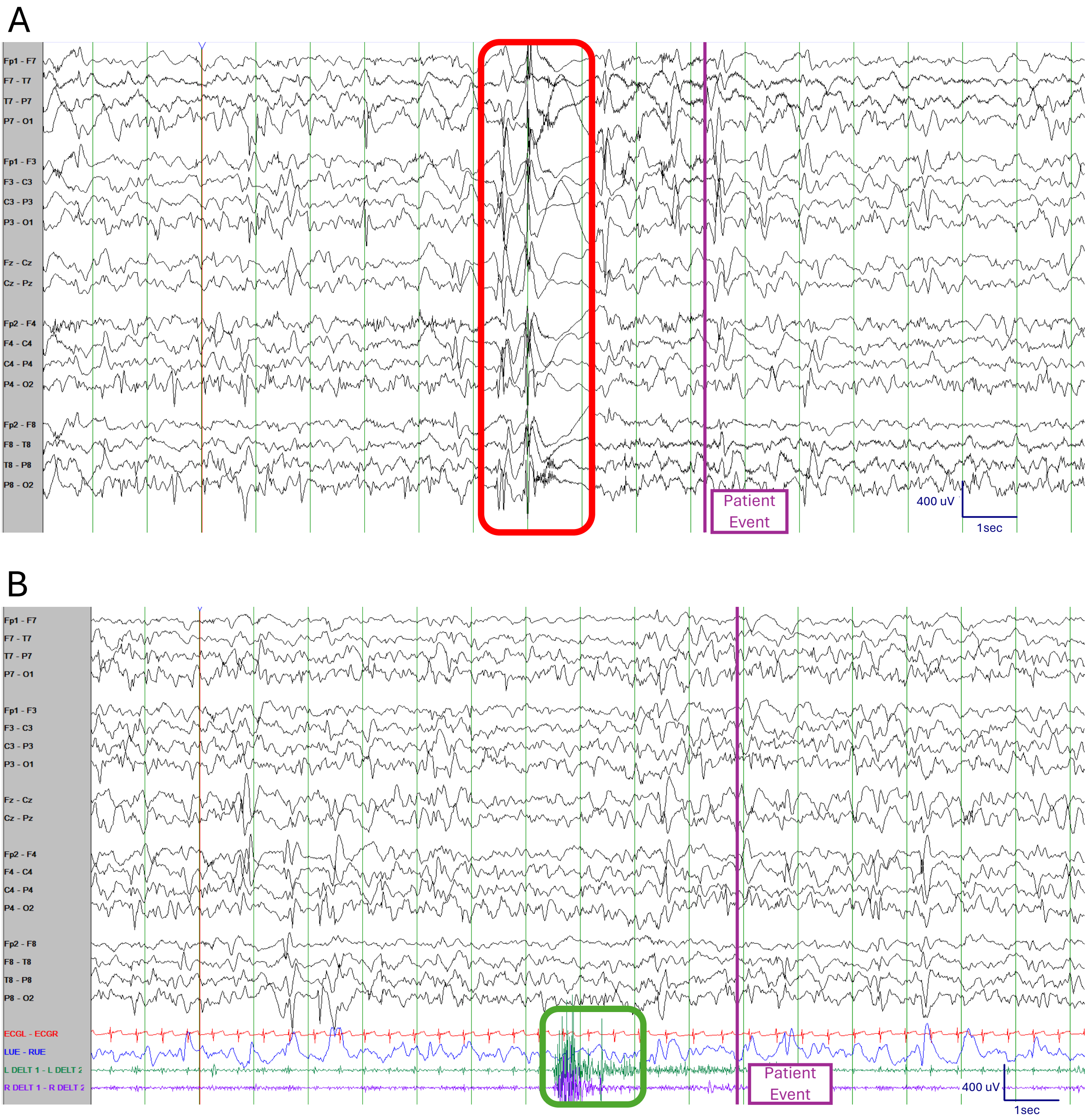

<u>Figure S1: Example of epileptic and non-epileptic myoclonus in Patient #1.</u>
A) A rapid body jerk is time-locked with generalized spike-wave discharge (red box), consistent with myoclonic seizure. **B)** Activation is seen in the EMG leads (green box) with associated patient event button, but no preceding epileptiform discharge in keeping with non-epileptic myoclonus. (AP bipolar montage, LFF 1 Hz, HFF 70 Hz, notch off, sensitivity 15 uV/mm, timebase 30 mm/sec)
