# Supplementary material for: The spectrum of movement disorders in young children with ARX ‐related epilepsy‐dyskinesia syndrome
Source: Ann Clin Transl Neurol. 2024 May 6;11(6):1643–7. doi: 10.1002/acn3.52055 (PMC11187834; doi:10.1002/acn3.52055)
Supplement: Supplementary file 2 — Figure S2. [file ACN3-11-1643-s005.pdf]

Supplementary Figure 2 – Patient 2 Representative EEG Panel

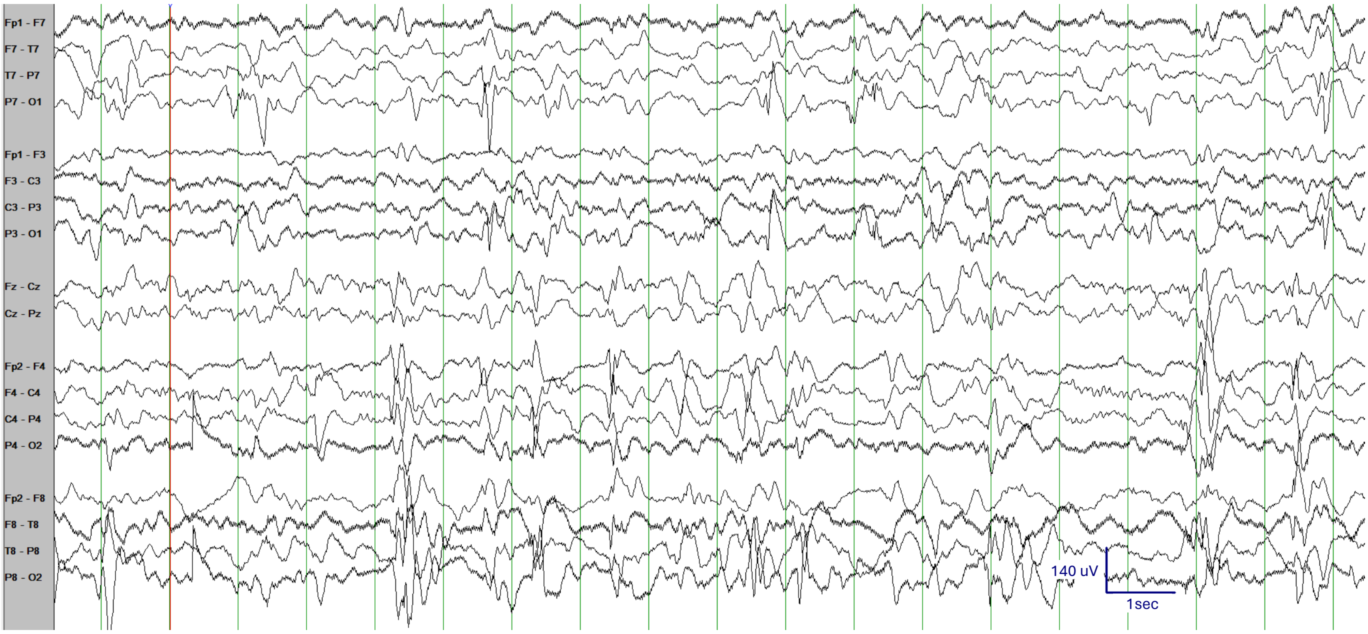

<u>Figure S2: Example of inter-ictal EEG in Patient #2.</u>

Abundant sleep-potentiated, high-voltage multifocal epileptiform discharges, more prominently over the right hemisphere, and poor background organization with absence of normal features of stage II sleep (AP bipolar montage, LFF 1 Hz, HFF 70 Hz, notch off, sensitivity 7 uV/mm, timebase 30 mm/sec)
