# Supplementary material for: The spectrum of movement disorders in young children with ARX ‐related epilepsy‐dyskinesia syndrome
Source: Ann Clin Transl Neurol. 2024 May 6;11(6):1643–7. doi: 10.1002/acn3.52055 (PMC11187834; doi:10.1002/acn3.52055)
Supplement: Supplementary file 3 — Figure S3. [file ACN3-11-1643-s003.pdf]

Supplementary Figure 3 – Patient 3 Representative EEG Panels

A

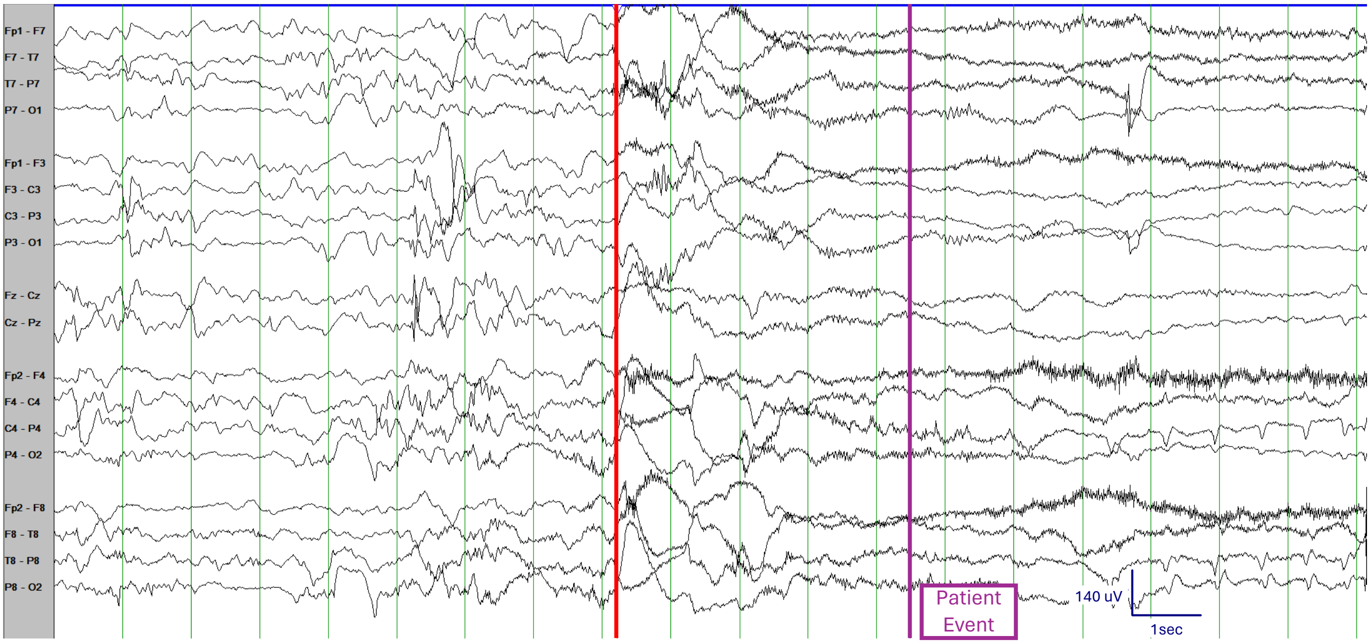

B

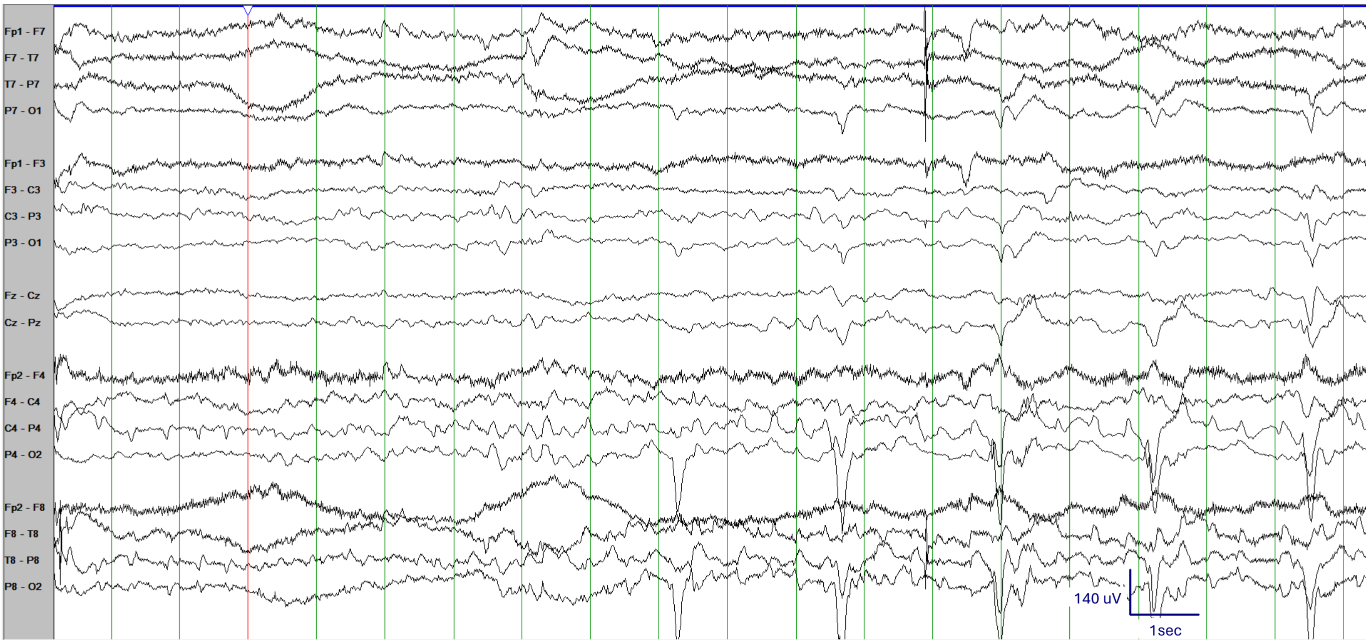

Continued Next Page ➔

C

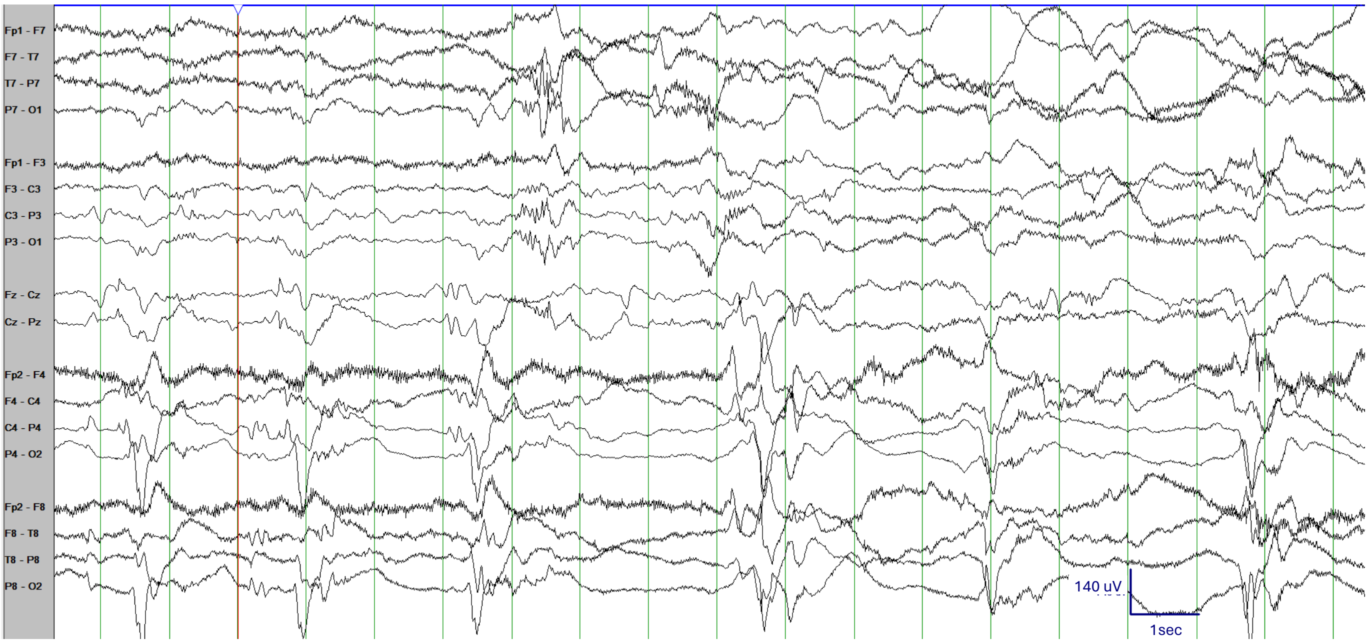

<u>Figure S3: Example of a tonic seizure followed by cluster of focal spasms in Patient #3.</u> **A)** There is a generalized slow wave (red vertical line) followed by diffuse attenuation with over-riding fast activity, then emergence of 1-2 Hz periodic spikes in the right centro-parieto-temporal region which **B)** evolves to 0.25-0.5 Hz periodic sharply contoured activity in the right occipital region. **C)** This further progresses to right hemispheric (posterior maximum) slow wave with overriding fast activity occurring every 2-8 seconds. Clinically, there is sudden extension of the arms and body stiffening followed by head and eye deviation to the right; the semiology then transitions to spasms with head and torso deviation to the left associated with arm extension and a gurgling sound. (AP bipolar montage, LFF 1 Hz, HFF 70 Hz, notch off, sensitivity 7 uV/mm, timebase 30 mm/sec)
